# Neuroanatomical correlates of distracted straight driving performance: a driving simulator MRI study across the lifespan

**DOI:** 10.3389/fnagi.2024.1369179

**Published:** 2024-04-19

**Authors:** Dylan X. Guan, Nathan W. Churchill, Corinne E. Fischer, Simon J. Graham, Tom A. Schweizer

**Affiliations:** ^1^Hotchkiss Brain Institute, University of Calgary, Calgary, AB, Canada; ^2^Neuroscience Research Program, St. Michael’s Hospital, Toronto, ON, Canada; ^3^Keenan Research Centre for Biomedical Science, St. Michael’s Hospital, Toronto, ON, Canada; ^4^Department of Physics, Toronto Metropolitan University, Toronto, ON, Canada; ^5^Department of Psychiatry, Faculty of Medicine, University of Toronto, Toronto, ON, Canada; ^6^Department of Medical Biophysics, University of Toronto, Toronto, ON, Canada; ^7^Hurvitz Brain Sciences Program, Sunnybrook Research Institute, Toronto, ON, Canada; ^8^Physical Sciences Platform, Sunnybrook Research Institute, Toronto, ON, Canada; ^9^Faculty of Medicine (Neurosurgery), University of Toronto, Toronto, ON, Canada

**Keywords:** magnetic resonance imaging, distracted driving, driving simulator, driving, aging

## Abstract

**Background:**

Driving is the preferred mode of transportation for adults across the healthy age span. However, motor vehicle crashes are among the leading causes of injury and death, especially for older adults, and under distracted driving conditions. Understanding the neuroanatomical basis of driving may inform interventions that minimize crashes. This exploratory study examined the neuroanatomical correlates of undistracted and distracted simulated straight driving.

**Methods:**

One-hundred-and-thirty-eight participants (40.6% female) aged 17–85 years old (mean and SD = 58.1 ± 19.9 years) performed a simulated driving task involving straight driving and turns at intersections in a city environment using a steering wheel and foot pedals. During some straight driving segments, participants responded to auditory questions to simulate distracted driving. Anatomical T1-weighted MRI was used to quantify grey matter volume and cortical thickness for five brain regions: the middle frontal gyrus (MFG), precentral gyrus (PG), superior temporal cortex (STC), posterior parietal cortex (PPC), and cerebellum. Partial correlations controlling for age and sex were used to explore relationships between neuroanatomical measures and straight driving behavior, including speed, acceleration, lane position, heading angle, and time speeding or off-center. Effects of interest were noted at an unadjusted *p*-value threshold of 0.05.

**Results:**

Distracted driving was associated with changes in most measures of straight driving performance. Greater volume and cortical thickness in the PPC and cerebellum were associated with reduced variability in lane position and heading angle during distracted straight driving. Cortical thickness of the MFG, PG, PPC, and STC were associated with speed and acceleration, often in an age-dependent manner.

**Conclusion:**

Posterior regions were correlated with lane maintenance whereas anterior and posterior regions were correlated with speed and acceleration, especially during distracted driving. The regions involved and their role in straight driving may change with age, particularly during distracted driving as observed in older adults. Further studies should investigate the relationship between distracted driving and the aging brain to inform driving interventions.

## 1 Introduction

Driving is a common everyday task for many individuals and the most frequently used mode of transportation in North America (Kim and Ulfarsson, 2004; Páez et al., 2007; Scott et al., 2009; Sleightholm et al., 2010). Despite this, motor vehicle crashes (MVCs) are among the leading causes of death in the United States. Driving is particularly dangerous for drivers at both ends of the adult age span, relative to middle-aged adults (Murphy et al., 2013; Cicchino and Mccartt, 2014). Young adults are more likely to be involved in fatal MVCs than any other age group (National Center for Statistics and Analysis, 2021), potentially due to inexperience and still-developing frontal brain regions that may promote risky driving behaviors (Williams, 2003). Older adults similarly face elevated risk of MVC-related injury and death (Meuleners et al., 2006; Cicchino and Mccartt, 2014), due to age-and health-related declines in the psychomotor skill and cognitive domains that support safe driving (Leversen et al., 2013; Robertsen et al., 2022). Physicians and governments should aim to minimize the risk of MVCs for adults across the age span (Carr et al., 2006), which requires the identification of high-risk drivers and driving behaviors. However, it remains an ongoing challenge to develop accurate and reliable tools for assessing fitness to drive, even within a single age cohort (Eby and Molnar, 2009; Eby and Molnar, 2010).

One of the difficulties in assessing driving fitness is the complexity of the driving task, which requires dynamic coordination of perceptual-motor skills and several cognitive domains, including executive function, attention, and decision-making, over time and across a wide array of situations (Anstey et al., 2005; Ranchet et al., 2012). Therefore, a simple assessment of key driving skills is useful, but insufficient in many cases. One important instance whereby cognitive capabilities can profoundly impact driving performance is during distracted driving, which requires a driver to engage simultaneously in a secondary task that competes for limited cognitive resources. Up to half of drivers across North America and Europe self-report having engaged in distracted driving (Woods-Fry et al., 2018; Lyon et al., 2021), yet distracted driving has been linked to delayed hazard detection (Harbluk et al., 2007), poorer visual scanning of the periphery (Engström et al., 2005), and an increased risk of MVCs for both novice and experienced drivers (Klauer et al., 2014). Such distraction-related driving impediments may pose a significant risk during simple maneuvers, such as straight driving - due to the difficulties in remaining vigilant during low cognitive demand, and the potential for “mind-wandering.” Furthermore, MVCs may be more severe during distracted straight driving, as straight driving permits higher speeds that considerably increase the risk of serious injuries and casualties (Elvik et al., 2004). These issues are expected to be exacerbated with age, as studies of seniors show greater compromise of cognitive capacity during motor tasks, when in the presence of auditory distraction (Da et al., 2015; Nieborowska et al., 2018; Bruce et al., 2019). Achieving a better understanding of the cognitive processes that underlie straight driving behaviors, and how they may be impaired by distracted driving across the healthy age span, is necessary to inform new methods for assessing driving fitness and to inform new strategies (e.g., cognitive interventions, assistive driving technology) to reduce the risk of severe MVCs.

The use of driving simulators combined with neuroimaging technology has emerged as an ecologically valid, reliable, and practical method to gain insight into the neural substrates of driving behavior (Walter et al., 2001; Calhoun et al., 2002; Uchiyama et al., 2003; Shechtman et al., 2009; Mayhew et al., 2011). This is bolstered by a substantial body of work assessing behavioral performance in driving simulations (Guo et al., 2019), including a growing number of studies showing that simulator tasks can provide valid representations of real-world driving across the age span (Wynne et al., 2019). In contrast to standardized on-road assessments, driving simulators allow for assessments of driving capacity under simple as well as more challenging scenarios that would otherwise put drivers, passengers, and other nearby road users at risk. For example, driving simulators have been used to evaluate driving fitness in individuals with neurological disorders, as well as healthy individuals under the influence of alcohol or engaged in distracted driving (Lew et al., 2005; Meda et al., 2009; Schweizer et al., 2013). Despite their utility, however, most neuroimaging studies of simulated driving possess several limitations. Study samples have generally consisted of adults from a single age group, most commonly young adults (Walter et al., 2001; Graydon et al., 2004) and rarely older adults (Hirth et al., 2007), despite observed differences in driving behavior across the lifespan (De Waard et al., 2009; Romoser et al., 2013). Sample sizes have tended to be small (<30 participants) and, in some cases, the driving simulator apparatus has had less ecological validity than desirable (Uchiyama et al., 2003; Hirth et al., 2007; Ohata et al., 2022). Most relevant to the present work, neuroimaging studies of simulated driving have focused predominantly on the patterns of brain activity evoked by different aspects of driving – leaving a large knowledge gap in understanding of how many other aspects of brain structure and physiological function may affect driving performance. Given that aging is tightly linked to neuroanatomical brain changes that may impact driving (Schultheis and Manning, 2011; Leversen et al., 2013; Bae et al., 2017) and that neuroanatomy forms the scaffold necessary to support the complexities of networked brain function, studies that relate brain structure to measures of simulated driving performance are strongly needed.

Consequently, the present study combined anatomical magnetic resonance imaging (MRI) with a virtual reality (VR) driving simulator to explore how two measures of grey matter tissue - volume and cortical thickness – were associated with various measures of distracted and undistracted driving performance in licensed drivers across the full adult age span. This setup was previously used by our group, in combination with functional MRI, to identify brain regions activated during different driving maneuvers in a cohort of young adults (Schweizer et al., 2013). They identified a broad network of driving-related areas; this included prefrontal, temporal, parietal and cerebellar regions. The present study extends this work by examining correlations of straight driving performance with neuroanatomy in a larger cohort that extends across the aging span of 17 to 85 years.

For this study, five brain regions of interest (ROIs) were selected *a priori* based on functional neuroimaging studies. These included the middle frontal gyrus (MFG), precentral gyrus (PG), posterior parietal cortex (PPC), superior temporal cortex (STC), and cerebellum. Activity in these regions was reliably identified in fMRI analyses using the same simulator apparatus (Schweizer et al., 2013). Moreover, activity in the MFG, PG, PPC, and cerebellum have been previously associated with driving speed (Calhoun et al., 2002). Driving under higher cognitive workloads and distracted driving have also been linked to activity in the MFG (Chung et al., 2014; Geissler et al., 2021) and in the STC (Choi et al., 2017). The relationship between grey matter volume/thickness and driving behaviors was assessed during straight driving segments, with and without auditory distraction. The moderating effect of age on brain-behavior relationships was also assessed. It was hypothesized that the ROIs would show a positive relationship between grey matter and measures of driving performance, based on literature evidence of a generally positive relationship between grey matter and cognitive function in adults (Westlye et al., 2011; Burzynska et al., 2012; Vonk et al., 2019). These relationships were also expected to be more pronounced in older adults, based on literature evidence of a stronger positive relationship between grey matter and cognitive function in older cohorts (De Chastelaine et al., 2019), potentially driven by heterogeneous declines in volume/thickness with age (Macdonald and Pike, 2021). Given the novelty of this study design, the above hypotheses were assessed within an exploratory analysis framework.

## 2 Materials and methods

### 2.1 Participant recruitment

A total of 138 participants, aged 17–85 years old, were recruited using advertisements and emails from the local community and university networks. Participants were required to be right-handed with normal or corrected vision and to self-identify as currently active drivers, i.e., holding a valid driver’s license and able to drive as needed. Participants were excluded if they had a history of psychiatric illness (e.g., bipolar disorder, major depressive disorder, post-traumatic stress disorder) or neurological illness (e.g., stroke, Alzheimer’s disease, Parkinson’s disease), were unable to undergo MRI (e.g., due to claustrophobia or ferromagnetic medical implants), had a history of alcohol of alcohol or drug abuse, or other diseases that may have altered blood flow significantly (e.g., cardiovascular diseases, kidney diseases, diabetes). Written informed consent was obtained from all participants prior to their inclusion in the study. Ethics approval was obtained by the Research Ethics Board at St. Michael’s Hospital and Baycrest Hospital in Toronto, Canada.

### 2.2 Driving simulator task

The simulated driving task was implemented in virtual reality (VR) using standard simulator software (STISIM Drive^®^). Participants lay supine in the MRI scanner and performed simulated driving using a steering wheel integrated with two additional response buttons, and using foot pedals for the accelerator and brake. All VR peripheral devices were MRI-compatible (Kan et al., 2013). Participants viewed the simulation through a mirror attached to the MRI head coil, which was oriented to reflect a projection screen. The screen was illuminated by an LCD system projecting through a waveguide in the radiofrequency shield of the MRI room. Participants also wore MRI-compatible headphones to hear the audio tasks.

Participants were asked to complete an hour-long training session outside of the MRI system before performing two runs of the driving task during MRI, when the image and driving performance data were recorded for data analysis. The training session reduced the effects of task acclimation, and mitigated risk of simulator sickness. Participants were briefed about possible issues and instructed to halt the experiment if they were in discomfort; a post-MRI debriefing session also assessed for issues. None of the participants experienced significant symptoms. The driving task was designed to simulate the standard licensing road test in the province of Ontario, Canada, requiring and required participants to perform straight driving segments interleaved with left and right turns at controlled intersections, all within a city environment. The posted speed limit throughout the entire task was 60 km/h. Straight driving segments included all time intervals (after initial acceleration at the start of the driving simulator run) that did not include turning or stopping maneuvers. During several straight driving sub-intervals, participants were presented with true-or-false questions, which they were required to answer using the response buttons. This served as an auditory distraction, similar to what would be expected from use of hands-free mobile devices (Lyon et al., 2021). The task runs consisted of 15 (run 1) and 14 (run 2) straight driving segments, broken up by the turn events. Only three straight driving segments per run included auditory distraction, to minimize habituation effects. Distracting events were also spread non-uniformly across runs to avoid anticipation. Ordering was fixed across participants, prioritizing the mitigation of variance over true randomization, with events in straight driving segments 3, 8, 13 (run 1) and 2, 8, 10 (run 2).

Measures of driving performance (see Table 1) were obtained using the STISIM Drive® software, including time elapsed (min), distance travelled (m), driving speed (km/h), lateral lane position (m), vehicle heading angle (°), crash events, and button press responses. Crash events were defined as participants colliding with cars or other objects during the driving task. Additional measures of driving performance were also generated from these data, including instantaneous acceleration, time spent speeding, time spent off-center, and the net number of correct responses to auditory distraction questions. Summary measures, including means, standard deviations (SDs), minimums, maximums, and sums were generated for each of these driving measures, as appropriate. All straight driving measures excluded segments that contained errors, collisions, and early task termination. The total number of driving errors and collisions across the entire task was also recorded and analyzed separately.

**Table 1:** Behavioral driving performance measures generated from driving simulator.

Variable	Description
Time elapsed	Time spent in driving condition in minutes (min)
Distance travelled	Distance covered in driving condition in meters (m)
Speed	Instantaneous speed in kilometers per hour (km/h)
Lateral lane position	Displacement from the lane center in meters (m)
Heading angle	Direction in which the vehicle is pointing in degrees (^o^)
Crashes	Number of collisions throughout entire task
Acceleration	Finite differences in instantaneous speed in kilometers per hour per second (km/h/s)
Time speeding	Percentage of total time in a driving condition in which the vehicle speed is >60 km/h (%)
Time off-center	Percentage of total time spent in a straight driving condition in which the vehicle is off-center >0.5 meters (%)
Net correct responses	Net sum of correct button presses during an auditory distraction condition with a score of +1 for correct responses, 0 for no responses, and − 1 for incorrect responses

Time elapsed, distance travelled, speed, lateral lane position, heading angle, and crashes were collected directly from the driving simulator software. Acceleration, time speeding, time off-center, and the net number of correct responses to auditory distraction questions were derived from the driving simulator outputs.

### 2.3 MR image acquisition and processing

All participants were imaged at St. Michael’s Hospital in Toronto, Canada using a 3.0 Tesla MRI system (Magnetom Skyra, Siemens, Erlangen, Germany) with a standard 20-channel head receiver coil. T1-weighted anatomical images were obtained using a Magnetization Prepared Rapid Acquisition Gradient Echo (MPRAGE) sequence: field-of-view (FOV) = 24 × 24 cm, 240 × 240 × 192 matrix, 0.9 mm isotropic voxels, bandwidth (BW) = 250 Hz/Pixel, inversion time (TI)/echo time (TE)/repetition time (TR) = 850/2.63/2000 ms, flip angle (FA) = 8°. All images were manually inspected for anatomical abnormalities and imaging artifacts before being run through an anatomical preprocessing pipeline using FreeSurfer version 6.0.^1^ This pipeline performed image registration into Talairach space, skull stripping, volumetric labeling, intensity normalization, white matter segmentation, surface atlas registration, surface extraction, gyral labeling, and subcortical segmentation (Fischl et al., 2002; Fischl, 2012). The FreeSurfer pipeline was executed on the Canadian Brain Imaging Research Platform (CBRAIN), a collaborative web-based research platform that provides access to High Performance Computing centers for computational analyses (Sherif et al., 2014). After preprocessing, each imaging dataset underwent a second round of quality control assessments for segmentation and grey and white matter surface labeling errors. Images with errors were manually edited to correct the error and then reprocessed locally. Using this approach, measures of grey matter volume and cortical thickness were generated for 34 cortical regions according to the Desikan-Killiany cortical atlas (Desikan et al., 2006), and measures of only grey matter volume were generated for 18 subcortical structures including the cerebellum according to the Automatic Segmentation of Subcortical Structures (Fischl et al., 2002) in each hemisphere.

### 2.4 Statistical analysis

For this study, five brain regions of interest (ROIs) were selected *a priori* based on previous functional neuroimaging studies that identified correlations between brain activity and different aspects of driving behavior. These ROIs included the middle frontal gyrus (MFG), precentral gyrus (PG), posterior parietal cortex (PPC), superior temporal cortex (STC), and cerebellum. Bilateral ROI values were generated by summing measures of grey matter volume or cortical thickness for the ROI in each hemisphere. To account for individual differences in head size, volumetric measures were divided by the total estimated intracranial volume, whereas thickness measures were not adjusted. Relevant outcome measures of driving performance included speed, acceleration, lane position, heading angle, number of crash events, time spent speeding, time off-center, and the number of correct responses to auditory distraction questions (Table 2). These measures were averaged across the two driving simulator runs for each participant to improve measurement reliability, during times of undistracted or distracted straight driving as indicated above.

**Table 2:** Driving simulator performance measures in undistracted *vs* distracted straight driving.

Variable	Straight driving	Distracted straight driving	*p*-value
Mean Speed (km/h)	48.1 ± 7.5 (26.2–61.2)	52.5 ± 9.1 (27.8–79.2)	<0.001^a^
SD Speed (km/h)	11.6 ± 3.3 (4.9–25.9)	7.7 ± 4.5 (0.5–23.6)	<0.001^a^
Mean Acceleration (km/h/s)	0.8 ± 0.3 (0–1.7)	-0.1 ± 0.6 (−1.5–1.4)	<0.001^b^
SD Acceleration (km/h/s)	2.7 ± 0.9 (1.2–6.6)	1.6 ± 1.2 (0.2–8.0)	<0.001^a^
Mean Lane Position (m)	2.6 ± 0.3 (1.8–3.5)	2.7 ± 0.5 (1.5–4.0)	<0.001^a^
SD Lane Position (m)	0.9 ± 0.6 (0.4–4.6)	0.6 ± 0.5 (0.1–3.4)	<0.001^a^
Mean Heading Angle (^o^)	0 ± 0.4 (−2.4–1.6)	0.1 ± 0.7 (−1.5–5.4)	0.005^1a^
SD Heading Angle (^o^)	2.4 ± 2.9 (0.6–22.1)	1.4 ± 2.2 (0.1–15.3)	<0.001^a^
Time Speeding (%)	11.7 ± 10.9 (0–54.1)	22.9 ± 34.6 (0–100)	0.334^a^
Time Off-Center (%)	67.4 ± 17.5 (11.7–93.1)	55.5 ± 32.2 (0–100)	0.002^a^

All variables are shown in mean ± standard deviation (range). Values were rounded to one decimal place except for p-values which were rounded to three decimal places.

a Paired samples Wilcoxon test.

b Paired samples *t*-test.

To control for the effects of baseline straight driving performance, we generated a distracted-undistracted contrast (DC) condition by subtracting the undistracted straight driving variable from the corresponding distracted straight driving variable. Prior to analysis, the distributions of each variable were visually assessed using histograms and boxplots for continuous variables and bar plots for categorical variables. Where appropriate, outliers were controlled for a given variable using 90% winsorization (i.e., 5th and 95th percentile thresholds). Measures of driving performance were then compared across undistracted and distracted straight driving conditions using paired samples t-tests or paired samples Wilcoxon tests, using the latter test when the paired differences were not normally distributed according to a Shapiro–Wilk normality test (*p*<0.05). Partial correlations were used to determine the association between the grey matter volume or cortical thickness of selected ROIs and measures of driving performance, while controlling for the effects of age and sex. Finally, age-dependent effects were evaluated by conducting partial correlations between ROI neuroanatomical measures and age-by-driving interaction terms. For interaction effects, the evolution of brain-behavior relationships across the aging span was illustrated by calculating the simple slopes correlation between the ROI measure and driving performance measure for three representative age values: low age (mean-1SD; 38 yrs.), middle age (mean; 58 yrs.) and high age (mean + 1SD; 78 yrs.); this visualization approach is commonly used in moderation analyses (Hayes and Rockwood, 2017). Regression diagnostics were conducted to ensure correlations were valid, including tests of linearity, normality of residuals, homogeneity of variances and tests of influence. Given the novelty of the data and the complex nature of the driving behavior, this study employed an exploratory approach in which all associations of potential interest were identified using an uncorrected statistical significance threshold of *p* < 0.05. All analyses were conducted on R version 4.0.2 (R Core Team, 2020). The raw data supporting the conclusions of this article will be made available by the authors upon request to interested researchers. The datasets analyzed for this study can be found in the figshare data repository at: https://doi.org/10.6084/m9.figshare.25546807.

## 3 Results

### 3.1 Participant characteristics and driving behavior

Participants (40.6% female) had a mean and standard deviation (SD) age of 58.1 ± 19.8 years (range = 17–85 years). Each driving simulator run lasted a total of 11.2 ± 1.6 min (range = 5.1–15.4 min). Participants spent 52.7 ± 5.2% (range = 34.0–64.2%) of their simulator run time performing undistracted straight driving and 3.8 ± 3.2% (range = 1.0–25.6%) performing distracted straight driving. The remainder of the simulator run time was spent preparing for or executing various turns at controlled intersections. Crash events were rare, occurring an average of 0.4 ± 0.9 (range = 0–6) times for each participant across both simulator runs, with 27.5% (*N* = 38) participants having at least one crash event over both runs.

A summary of driving performance measures obtained for undistracted and distracted straight driving conditions is listed in Table 2. During the distracted condition, participants tended to drive faster and maintain a lane position farther from the center line, relative to the undistracted condition. However, during the distracted condition, participants also showed lower speed variability, lower average acceleration, more time off-center, and less variability in lane position, heading angle, and acceleration relative to the distracted condition. Although participants spent twice as much time, on average, speeding during distracted driving compared to undistracted driving, this difference was not significant due to high inter-individual variability, particularly during the distracted condition.

### 3.2 Brain-driving correlations

**Figure 1:**
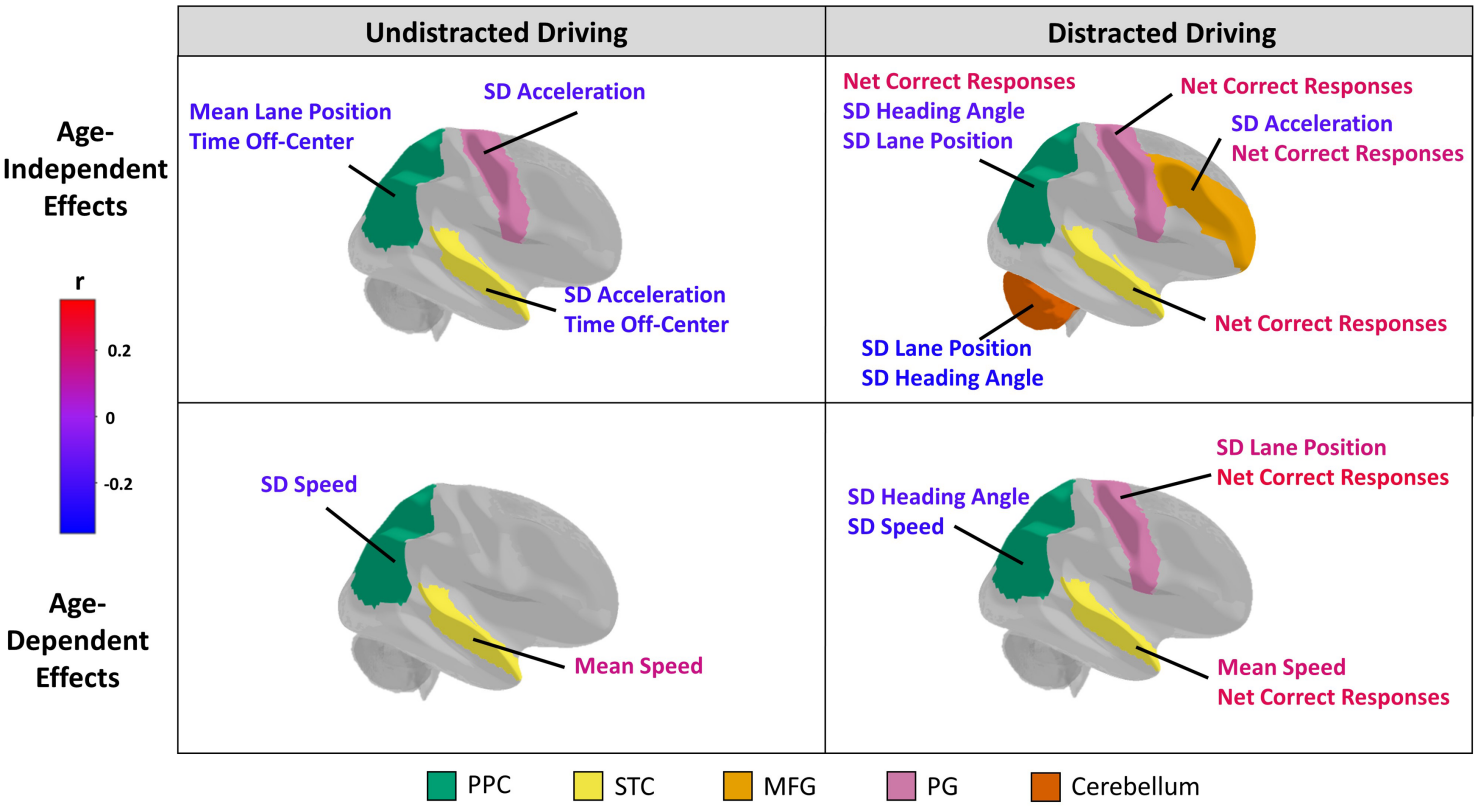
Structural neural correlates of driving performance. The five regions of interest selected for analysis are color-coded and include the posterior parietal cortex (PPC; green), superior temporal cortex (STC; yellow), middle frontal gyrus (MFG; light orange), precentral gyrus (PG; pink), and cerebellum (orange). Brain regions are plotted showing Age-Independent (top row) and Age-dependent (bottom row) associations with driving, with effects plotted separately for Undistracted (left) and Distracted (right) behaviors. For each category, brain regions showing at least one effect at *p* < 0.05 (unadjusted) are coloured in. The associated behavioral indices are also labelled for these regions. The color of the labels indicate the direction and strength of a correlation between a driving behavioral measure (for age-independent effects) or driving behavioral measure*age interaction term (for age-dependent effects) and the volume or thickness of a region of interest according to a color bar. Specifically, a red or blue age-dependent effect indicates that the relationship becomes more positive or more negative, respectively, with age. The orientation for all brain figures run from posterior (left) to anterior (right). Although only the right brain hemispheres are shown, grey matter volume or cortical thickness measures of bilateral regions of interests were analyzed.

All associations between straight driving performance measures and ROI grey matter volume and cortical thickness that reached the nominal threshold of *p* < 0.05 are summarized in Figure 1; see Appendix-1 for the complete report of all analyses conducted in this study. Partial correlation coefficients, 95% confidence intervals, and *p*-values can be found in Table 3 for main effects and Table 4 for age-dependent effects, along with simple slopes correlations at representative low, middle, and high levels of age. Grey matter volumes in only the PPC and cerebellum were associated with driving measures at an uncorrected *p*<0.05. Participants with greater PPC volume tended to spend less time off-center during undistracted straight driving, and demonstrated reduced variability in lane position and heading angle in the DC (i.e., distracted-undistracted contrast) condition. Similarly, greater cerebellum volume was associated with reduced variability in lane position and heading angle during distracted straight driving. Cortical thickness measures in the MFG, PCG, PPC, and STC were all associated with at least one driving measure at an uncorrected *p* < 0.05. Across all four ROIs, greater cortical thickness was associated with a greater number of correct responses to the auditory distraction questions. For the PG and STC, these associations with the number of correct responses were age-dependent, such that the associations became more positive and stronger with age. Participants with greater MFG thickness tended to show lower variability in acceleration during distracted straight driving, whereas greater PG and STC thicknesses were associated with lower variability in acceleration during undistracted straight driving. Those with greater STC thickness also tended to spend less time off-center.

**Table 3:** Partial correlations between driving performance measures and brain region volume and thickness.

ROI	*r*	95% CI	*p*-value
*Volume*			
Posterior Parietal Cortex			
Mean Lane Position (US)	−0.20	−0.36 – −0.03	0.026
Time Off-Center (US)	−0.21	−0.38 – −0.05	0.016
SD Lane Position (DC)	−0.17	−0.34 – −0.01	0.049
SD Heading Angle (DC)	−0.17	−0.34 – −0.01	0.048
Cerebellum			
SD Lane Position (DS)	−0.27	−0.42 – −0.11	0.003
SD Heading Angle (DS)	−0.28	−0.44 – −0.12	0.002
*Thickness*			
Middle Frontal Gyrus			
SD Acceleration (DS)	−0.18	−0.35 – −0.02	0.040
Net Correct Responses	0.19	0.02–0.35	0.032
Precentral Gyrus			
SD Acceleration (US)	−0.18	−0.35 – −0.02	0.039
Net Correct Responses	0.21	0.05–0.37	0.015
Posterior Parietal Cortex			
Net Correct Responses	0.21	0.05–0.37	0.016
Superior Temporal Cortex			
SD Acceleration (US)	−0.21	−0.37 – −0.05	0.017
Time Off-Center (US)	−0.21	−0.37 – −0.05	0.018
Net Correct Responses	0.23	0.07–0.40	0.007

Partial correlations controlled for age and sex. Net correct responses were determined by subtracting the number of incorrect responses from the sum of correct responses across an entire driving simulator run and then averaged across both driving simulator runs per participant. All values have been rounded to two decimal places except for p-values which were rounded to 3 decimal places. ROI, region of interest; 95% CI, 95% confidence interval; DC, distraction-undistracted contrast; US, undistracted straight driving; DS, distracted straight driving; SD, standard deviation.

**Table 4:** Age-dependent relationships between driving performance and brain volume and thickness.

	Low age		Middle age		High age		
ROI	*r*	95% CI	*r*	95% CI	*r*	95% CI	*p*-value
*Volume*							
Precentral Gyrus							
SD Lane Position (DC)	−0.23	−0.39 – −0.07	−0.19	−0.36 – −0.03	-0.03	−0.20 – 0.14	0.047
Net Correct Responses	−0.09	−0.25 – 0.08	0.04	−0.13 – 0.21	0.18	0.01–0.34	0.043
*Thickness*							
Precentral Gyrus							
Net Correct Responses	−0.06	−0.23 – 0.11	0.14	−0.03 – 0.30	0.31	0.15–0.46	0.008
Posterior Parietal Cortex							
SD Speed (US)	0.08	−0.09 – 0.25	−0.05	−0.22 – 0.12	−0.18	−0.35 – −0.02	0.035
SD Speed (DS)	0.14	−0.03 – 0.31	0	−0.17 – 0.17	−0.17	−0.34 – −0.01	0.011
SD Heading Angle (DS)	0.09	−0.08 – 0.26	−0.05	−0.22 – 0.12	−0.22	−0.38 – −0.05	0.041
Superior Temporal Cortex							
Mean Speed (US)	−0.09	−0.26 – 0.07	0.01	−0.16 – 0.18	0.19	0.03–0.35	0.041
Mean Speed (DS)	−0.11	−0.28 – 0.06	0	−0.17 – 0.17	0.18	0.02–0.35	0.027
Net Correct Responses	−0.03	−0.20 – 0.14	0.16	−0.01 – 0.33	0.32	0.17–0.47	0.011

All coefficients represent simple slopes correlation coefficients for the association between an ROI and driving measure, calculated for a set of fixed ages for illustrative purposes. They include low age (mean-1 SD; 38 yrs.), middle age (mean; 58 yrs.), and high age (mean + 1 SD; 78 yrs.). Net correct responses were determined by subtracting the number of incorrect responses from the sum of correct responses across an entire driving simulator run and then averaged across both driving simulator runs per participant. All p-values indicate the statistical significance of the age-by-driving interaction. All partial correlations controlled for age and sex. Values have been rounded to two decimal places except for p-values which have been rounded to three decimal places. ROI, region of interest; 95% CI, 95% confidence interval; DC, distraction-undistracted contrast; US, undistracted straight driving; DS, distracted straight driving; SD, standard deviation.

Some correlations between ROI neuroanatomical measures and straight driving behaviors only emerged in the context of aging. For instance, grey matter volume in the PG was negatively correlated with lane position variability in the DC condition for young adults, but this association weakened with age. In contrast, PG cortical thickness was weakly correlated with the number of correct responses to auditory questions during distracted straight driving in young adults, but this association became more positive and strengthened with age. Both associations failed to attain a threshold of *p* < 0.05 when modelled as a main effect alone (i.e., without its interaction with age). The cortical thicknesses of the PPC and STC also exhibited age-dependent effects on measures of straight driving performance pertaining to speed. Specifically, greater cortical thickness in the PPC was correlated with greater speed variability in young adults but lower speed variability in older adults during both undistracted and distracted straight driving. In contrast, younger adults with greater STC cortical thicknesses tended to drive at lower speeds whereas older adults with greater STC thicknesses tended to drive at higher speeds. Finally, cortical thickness in the PPC was also correlated with greater heading angle variability during distracted straight driving, but this association became increasingly more negative with age.

## 4 Discussion

This exploratory study measured the neuroanatomical neural correlates of straight driving behaviors, with and without auditory distraction. This was assessed in a sample of 138 participants across the full adult age span, by employing both MRI and a VR driving simulator. Multiple associations were identified between driving behaviors and neuroanatomical ROIs at an uncorrected *p*-value threshold of 0.05, including associations that were consistent across age and those that varied in an age-dependent manner. The study findings were broadly consistent with our initial hypotheses. Grey matter volume/thickness was correlated with position and speed maintenance (i.e., reduced SD of lane position, heading angle and acceleration), along with correct responses to auditory questions. This aligns with literature indicating a positive relationship between grey matter and cognitive task performance in adults (Westlye et al., 2011; Burzynska et al., 2012; Vonk et al., 2019). In addition, grey matter correlations with position and speed maintenance (i.e., reduced SD for speed and heading angle – but not for lane position), along with correct responses to auditory questions, were increased with age. These results are aligned with literature reporting a stronger positive relationship between grey matter and cognitive function in older cohorts (De Chastelaine et al., 2019). Collectively, the results of this study provide encouraging preliminary evidence of a neuroanatomical basis for individual differences in driving performance across the age span that is broadly congruent with prior studies of aging and cognition.

In this study, auditory distraction was linked to changes in nearly all behavioral measures of straight driving performance. Importantly, distracted driving was associated with higher driving speeds when compared to undistracted driving, which is known to increase the risk of severe injury and death during MVCs (Elvik et al., 2004). Such a change may result from the reallocation of cognitive or attentional resources away from driving and towards the secondary task, making it more difficult for participants to maintain a speed just below the speed limit (as they did during undistracted straight driving intervals). Consistent with this assertion, prior studies that combined simulated driving with functional neuroimaging found that the introduction of secondary tasks generally changed patterns of brain activity in participants, with increased frontal activity and decreased parietal activity (Just et al., 2008; Lin et al., 2011; Chung et al., 2014; Choi et al., 2017; Unni et al., 2017). In addition, the brain-driving associations that were relatively consistent across distracted and undistracted conditions in our study suggest a common neuroanatomical substrate for certain driving behaviors, irrespective of the presence or absence of distraction. This is discussed more below.

An earlier study employing the same driving simulator apparatus and experimental paradigm, but examining an independent sample of young adults, observed activation in several regions in the frontal, temporal, and parietal lobes during distracted straight driving (Schweizer et al., 2013). Similar results have been identified in other functional neuroimaging studies using driving simulators for undistracted straight driving (Walter et al., 2001; Calhoun et al., 2002; Uchiyama et al., 2003; Graydon et al., 2004; Fort et al., 2010). Collectively, these studies suggest that the dynamic coordination and recruitment of a wide and distributed network of brain regions is crucial for successful driving. However, because of the intrinsic methodology adopted, such studies have yet to provide a clear determination of which brain regions are functionally implicated, and which are merely “along for the ride.” The results of the present work help to address this question through another lens, showing that the PPC and cerebellum were correlated with measures of lane maintenance, such as lane position and heading angle variability, whereas nearly all ROIs including the MFG, PG, STC, and PPC were associated with measures of speed and acceleration. The PPC and cerebellum play critical roles in visuospatial perception and visuomotor and bimanual coordination, respectively (Thach et al., 1992; Andersen et al., 1997; Boisgontier et al., 2018), which may explain their role in lane maintenance. Greater neural resources in these regions may enable participants to perceive when they are failing to maintain lane position, and to subsequently make the appropriate steering wheel movements to correct lane position based on visual feedback. In contrast, driving speed and acceleration may involve a wider array of neural structures, including more anterior regions, because these areas are often engaged in the context of goals, integrating both endogenous and exogenous cues. The MFG is thought to reorient attentional resources from endogenous attentional networks to exogenous cues (Corbetta et al., 2008). Greater cortical thickness in the MFG may thus provide more resources for efficiently reorienting attention after attending to auditory instructions (which implicates the STC) and pressing the brake pedal to decelerate and prepare for the turn (which implicates the PG). This conceptual framework is supported by a few early functional neuroimaging studies of simulated driving that correlated brain activity to aspects of driving performance in smaller samples. Carvalho et al. (2006) found that speed control (braking) activated frontal motor areas while steering maintenance activated the cerebellum, and Calhoun et al. (2002) found that MFG activity was associated with driving speed. Altogether, the present study provides important independent evidence from anatomical neuroimaging that volume and cortical thickness of different brain regions are correlated with distinct measures of driving performance.

By testing the moderating effect of age on brain-behavior correlations, it was found that many of the observed associations between brain structure and driving behavior were age-dependent; and also that certain associations were not a main effect of behavior, but nevertheless emerged when analyzed as an age-by-behavior interaction. Increasing age tended to make the correlations between PPC thickness and speed variability more negative and the correlations between STC thickness and average speed more positive. In other cases, aging served to weaken the correlation, such between PG volume and lane position maintenance. These findings suggest that older adults may be recruiting brain regions in different ways to drive compared to their younger counterparts, with the impact of local neural resources on driving performance varying across the age span. This is likely due in part to changes in behavioral driving strategies as individuals grow older to compensate for the decline in certain skills or abilities that used to support earlier driving behaviors (Rabbitt et al., 1996; Hansen et al., 2020; Robertsen et al., 2022). For example, older drivers tend to merge into heavy traffic more slowly than younger drivers (De Waard et al., 2009), show differences in the number of eye movements and visual search strategies at intersections (Maltz and Shinar, 1999; Bao and Boyle, 2007; Romoser et al., 2013), and have slower driving-related reaction time (Leversen et al., 2013). Furthermore, these findings provide an indication of how neuroanatomical measures may relate to better or poorer driving performance across the adult lifespan. For example, larger neuroanatomical measurements of the PPC and cerebellum were linked to better speed and lane maintenance, and therefore better driving performance, across the entire cohort.

By including over 100 participants across the adult age span, the present study was able to explore brain-behavior associations with sufficient statistical power and in the context of age. The VR driving simulator employed in our study possessed relatively high ecological validity, as participants completed a driving task that was based on a provincial driving licensing exam, using a steering wheel and accelerator and brake foot pedals. The simulated distracted driving was also designed to approximate the most ecological and common form of distraction: using a mobile device for verbal communication (Lyon et al., 2021). Although requiring replication, these aspects support the generalizability of the present study findings across different age groups and to other settings, hopefully to inform real-world driving intervention and policy research. Our exploratory approach also helped to identify trends that may have otherwise been overlooked using a focused hypothesis-driven approach. Finally, the driving simulator software employed in this study, STISIM Drive®, provided a wealth of behavioral data that include both typical measures of driving behavior in the existing literature, such as speed, lane maintenance, and collisions (Calhoun et al., 2002; Carvalho et al., 2006; Just et al., 2008; Lin et al., 2011; Liu et al., 2012), as well as more comprehensive intra-task behavior such as acceleration and time spent speeding or off-center. Given the complexity of driving tasks, incorporating more comprehensive measures of driving behavior into future studies may generate novel and important insight into strategies to lower MVC risk.

The present study also had several experimental limitations. Strictly, the distracted and undistracted straight driving conditions were not perfectly controlled, so that their comparison purely isolated the effect of distraction. For example, undistracted straight driving could have occurred at the beginning of a simulator run or immediately after a turn, whereas distracted driving conditions generally commenced after the preferred speed had been reached. In each of these cases, carry-over effects from previous performance (or lack thereof) could have introduced confounds. Furthermore, the above conditions likely contributed to the observation that the average acceleration was lower during distracted driving than during undistracted driving, despite the opposite pattern occurring for average speed. The participant sample in this study was also skewed towards older and middle-aged adults; while the greater sample of upper ages helps to mitigate variance caused by heterogeneous aging, and reductions in the contrast-to-noise ratio (CNR) of T1-weighted images (Knight et al., 2016), this increases the leverage exerted by young adults and may lead to their greater influence in the regression models. Our regression diagnostics found no evidence that this was an issue, nevertheless, it may benefit future studies to obtain balanced samples across the aging span. In addition, the present exploratory approach reported effects at an unadjusted *p*-value threshold of 0.05. This relaxed thresholding approach was chosen in the present study given the paucity of research in this area, to avoid discarding potential effects of future interest but at the expense of an increased risk of false positives. We recommend using our findings to generate hypotheses for future simulated driving neuroimaging studies to test using confirmatory hypothesis-driven approaches.

Numerous questions about the relationship between the brain, driving, age, and distraction remain, raising interesting possibilities for future research. For example, the present study raises the question of how brain-behavior relationships change under different forms of distracted driving. Although our study focused on auditory distractions, other forms of distracted driving exist, such as texting while driving, and particularly in the context of social media. Distractions that involve a visual component may be of particular concern, given that dual-task literature shows greater interference for secondary tasks that engage similar neural circuitry as the primary one (Li et al., 2018). Such forms of distracted driving may require greater reallocation of cognitive resources (including social cognition), induce stronger changes in driving performance, and may increase the risk of causing MVCs even further. As the prevalence of distracted driving rises (Lyon et al., 2021), neuroimaging driving simulator studies investigating the impact of distracted driving are increasingly warranted. Participants in this study were not explicitly instructed in how to handle the auditory distraction task, to mimic real-world conditions in which distracted drivers must choose to prioritize driving or a secondary task. This raises further questions as to whether better performers can be divided into those who sacrifice performance on the auditory task in favour of driving and those who divide their attention successfully between tasks (Young et al., 2007), and whether these groups have distinct neural correlates. To address this question, future work should include a “baseline” assessment of auditory task performance, in order to assess subsequent decline during driving (or lack thereof). Finally, the present study focused on grey matter tissue, rather than white matter. This approach was chosen because grey matter regions can be linked to specific domains of function, and can be compared to prior functional neuroimaging analyses. Nevertheless, future work should consider white matter as well, since morphometric changes have been correlated with both aging and declines in cognitive function (Gunning-Dixon et al., 2009).

It also remains speculative how the measures of driving behavior used in present study relate to real-world driving outcomes. Although higher driving speeds and less consistent lane maintenance may reasonably be inferred to increase the risk of MVCs, studies are needed to show that an individual’s performance in a driving simulator predicts their real-world driving performance and outcomes. Further research is also needed to better understand how age-related changes in the brain affect driving fitness, and what interventions may be appropriate. Simply restricting the ability of older adults to drive is not a feasible solution to address these age-related changes in driving. Driving is an important source of autonomy and is often necessary to meet the daily transport needs of older adults (Oxley and Whelan, 2008; Hansen et al., 2020). Furthermore, revoking one’s driving license and ability to drive has been linked to several worse general health, psychological, and social outcomes (Chihuri et al., 2016; Litwin and Levinson, 2018; Nyberg et al., 2021). A better understanding of age-related changes in driving behavior and the neural substrates underlying those behaviors is necessary to support the ability of older adults to drive while simultaneously lowering the risk of MVCs. For instance, knowing that atrophy in certain brain regions in older adults is linked to deficits in specific aspects of driving may inform interventions, such as restricted or conditional licenses, that allow older drivers to continue driving but only within geographical areas or under conditions that are appropriate for their individual driving capacities. Such work may also help in the development of assistive “safe mode” driving tools and controls to enhance driving performance in certain individuals. To this end, future neuroimaging studies of driving should include participants in various age groups across the lifespan and should provide analyses in the context of aging.

## Data availability statement

The original contributions presented in the study are publicly available. This data can be found here: Figshare, https://doi.org/10.6084/m9.figshare.25546807.

## Ethics statement

The studies involving humans were approved by Research Ethics Board at St. Michael’s Hospital and Baycrest Hospital. The studies were conducted in accordance with the local legislation and institutional requirements. The participants provided their written informed consent to participate in this study.

## Author contributions

DG: Conceptualization, Data curation, Formal analysis, Visualization, Writing – original draft. NC: Conceptualization, Data curation, Software, Writing – review & editing. CF: Data curation, Investigation, Resources, Writing – review & editing. SG: Data curation, Investigation, Methodology, Resources, Writing – review & editing. TS: Conceptualization, Data curation, Funding acquisition, Investigation, Methodology, Resources, Supervision, Writing – review & editing.

## Footnotes

^1^ https://surfer.nmr.mgh.harvard.edu/

## References

[ref1] Andersen R. A. Snyder L. H. Bradley D. C. Xing J. (1997). Multimodal representation of space in the posterior parietal cortex and its use in planning movements. Annu. Rev. Neurosci. 20, 303–330. doi: 10.1146/annurev.neuro.20.1.303, PMID: 9056716

[ref2] Anstey K. J. Wood J. Lord S. Walker J. G. (2005). Cognitive, sensory and physical factors enabling driving safety in older adults. Clin. Psychol. Rev. 25, 45–65. doi: 10.1016/j.cpr.2004.07.008, PMID: 15596080

[ref3] Bae S.-H. Sabando J. F. Kim S.-H. (2017). Elder drivers and age-related changes: a user requirement analysis for in-vehicle information system. J. Korea Safety Manag. Sci. 19, 103–114. doi: 10.12812/ksms.2017.19.1.103

[ref4] Bao S. Boyle L. N. (2007). “Visual search strategies of older drivers at rural expressway intersections” in Proceedings of the human factors and ergonomics society annual meeting (Los Angeles, CA: SAGE Publications Sage CA), 1560–1564.

[ref5] Boisgontier M. P. Cheval B. Van Ruitenbeek P. Cuypers K. Leunissen I. Sunaert S.. (2018). Cerebellar gray matter explains bimanual coordination performance in children and older adults. Neurobiol. Aging 65, 109–120. doi: 10.1016/j.neurobiolaging.2018.01.016, PMID: 29471213

[ref6] Bruce H. Aponte D. St-Onge N. Phillips N. Gagné J.-P. Li K. Z. (2019). The effects of age and hearing loss on dual-task balance and listening. J. Gerontol. B Psychol. Sci. Soc. Sci. 74, 275–283. doi: 10.1093/geronb/gbx047, PMID: 28486677 PMC6327658

[ref7] Burzynska A. Z. Nagel I. E. Preuschhof C. Gluth S. Bäckman L. Li S. C.. (2012). Cortical thickness is linked to executive functioning in adulthood and aging. Hum. Brain Mapp. 33, 1607–1620. doi: 10.1002/hbm.21311, PMID: 21739526 PMC6869850

[ref8] Calhoun V. D. Pekar J. J. Mcginty V. B. Adali T. Watson T. D. Pearlson G. D. (2002). Different activation dynamics in multiple neural systems during simulated driving. Hum. Brain Mapp. 16, 158–167. doi: 10.1002/hbm.10032, PMID: 12112769 PMC6872105

[ref9] Carr D. B. Meuser T. M. Morris J. C. (2006). Driving retirement: the role of the physician. CMAJ 175:601. doi: 10.1503/cmaj.060971, PMID: 16966663 PMC1559414

[ref10] Carvalho K. N. Pearlson G. D. Astur R. S. Calhoun V. D. (2006). Simulated driving and brain imaging: combining behavior, brain activity, and virtual reality. CNS Spectr. 11, 52–62. doi: 10.1017/S1092852900024214, PMID: 16400256

[ref11] Chihuri S. Mielenz T. J. Dimaggio C. J. Betz M. E. Diguiseppi C. Jones V. C.. (2016). Driving cessation and health outcomes in older adults. J. Am. Geriatr. Soc. 64, 332–341. doi: 10.1111/jgs.13931, PMID: 26780879 PMC5021147

[ref12] Choi M.-H. Kim H.-S. Yoon H.-J. Lee J.-C. Baek J.-H. Choi J.-S.. (2017). Increase in brain activation due to sub-tasks during driving: fMRI study using new MR-compatible driving simulator. J. Physiol. Anthropol. 36, 1–12. doi: 10.1186/s40101-017-0128-8 PMC5270359 28126038

[ref13] Chung S.-C. Choi M.-H. Kim H.-S. You N.-R. Hong S.-P. Lee J.-C.. (2014). Effects of distraction task on driving: a functional magnetic resonance imaging study. Biomed. Mater. Eng. 24, 2971–2977. doi: 10.3233/BME-141117, PMID: 25227004

[ref14] Cicchino J. B. Mccartt A. T. (2014). Trends in older driver crash involvement rates and survivability in the United States: an update. Accid. Anal. Prev. 72, 44–54. doi: 10.1016/j.aap.2014.06.011, PMID: 25003969

[ref15] Corbetta M. Patel G. Shulman G. L. (2008). The reorienting system of the human brain: from environment to theory of mind. Neuron 58, 306–324. doi: 10.1016/j.neuron.2008.04.017, PMID: 18466742 PMC2441869

[ref16] Da H. K. Lee J. D. Lee H. J. (2015). Relationships among hearing loss, cognition and balance ability in community-dwelling older adults. J. Phys. Ther. Sci. 27, 1539–1542. doi: 10.1589/jpts.27.1539 26157259 PMC4483437

[ref17] De Chastelaine M. Donley B. E. Kennedy K. M. Rugg M. D. (2019). Age moderates the relationship between cortical thickness and cognitive performance. Neuropsychologia 132:107136. doi: 10.1016/j.neuropsychologia.2019.107136, PMID: 31288025 PMC6702041

[ref18] De Waard D. Dijksterhuis C. Brookhuis K. A. (2009). Merging into heavy motorway traffic by young and elderly drivers. Accid. Anal. Prev. 41, 588–597. doi: 10.1016/j.aap.2009.02.011 19393811

[ref19] Desikan R. S. Ségonne F. Fischl B. Quinn B. T. Dickerson B. C. Blacker D.. (2006). An automated labeling system for subdividing the human cerebral cortex on MRI scans into gyral based regions of interest. NeuroImage 31, 968–980. doi: 10.1016/j.neuroimage.2006.01.021, PMID: 16530430

[ref20] Eby D. W. Molnar L. J. (2009). Older adult safety and mobility: issues and research needs. Public Works Manag. Policy 13, 288–300. doi: 10.1177/1087724X09334494

[ref21] Eby D. W. Molnar L. J. (2010). Driving fitness and cognitive impairment: issues for physicians. JAMA 303, 1642–1643. doi: 10.1001/jama.2010.495 20424255

[ref22] Elvik R. Christensen P. Amundsen A. H. (2004). Speed and road accidents: an evaluation of the power model. Oslo, Norway: Transportøkonomisk Institutt.

[ref23] Engström J. Johansson E. Östlund J. (2005). Effects of visual and cognitive load in real and simulated motorway driving. Transport. Res. F: Traffic Psychol. Behav. 8, 97–120. doi: 10.1016/j.trf.2005.04.012

[ref24] Fischl B. (2012). FreeSurfer. Neuroimage 62, 774–781. doi: 10.1016/j.neuroimage.2012.01.021, PMID: 22248573 PMC3685476

[ref25] Fischl B. Salat D. H. Busa E. Albert M. Dieterich M. Haselgrove C.. (2002). Whole brain segmentation: automated labeling of neuroanatomical structures in the human brain. Neuron 33, 341–355. doi: 10.1016/S0896-6273(02)00569-X 11832223

[ref26] Fort A. Martin R. Jacquet-Andrieu A. Combe-Pangaud C. Foliot G. Daligault S.. (2010). Attentional demand and processing of relevant visual information during simulated driving: a MEG study. Brain Res. 1363, 117–127. doi: 10.1016/j.brainres.2010.09.094, PMID: 20920486

[ref27] Geissler C. F. Schneider J. Frings C. (2021). Shedding light on the prefrontal correlates of mental workload in simulated driving: a functional near-infrared spectroscopy study. Sci. Rep. 11, 1–10. doi: 10.1038/s41598-020-80477-w 33436950 PMC7804012

[ref28] Graydon F. X. Young R. Benton M. D. Genik R. J. Posse S. Hsieh L.. (2004). Visual event detection during simulated driving: identifying the neural correlates with functional neuroimaging. Transport. Res. F: Traffic Psychol. Behav. 7, 271–286. doi: 10.1016/j.trf.2004.09.006

[ref29] Gunning-Dixon F. M. Brickman A. M. Cheng J. C. Alexopoulos G. S. (2009). Aging of cerebral white matter: a review of MRI findings. Int. J. Geriatr. Psychiatry 24, 109–117. doi: 10.1002/gps.2087 18637641 PMC2631089

[ref30] Guo F. Lv W. Liu L. Wang T. Duffy V. G. (2019). Bibliometric analysis of simulated driving research from 1997 to 2016. Traffic Inj. Prev. 20, 64–71. doi: 10.1080/15389588.2018.1511896 30888870

[ref31] Hansen S. Newbold K. B. Scott D. M. Vrkljan B. Grenier A. (2020). To drive or not to drive: driving cessation amongst older adults in rural and small towns in Canada. J. Transp. Geogr. 86:102773. doi: 10.1016/j.jtrangeo.2020.102773

[ref32] Harbluk J. L. Noy Y. I. Trbovich P. L. Eizenman M. (2007). An on-road assessment of cognitive distraction: impacts on drivers’ visual behavior and braking performance. Accid. Anal. Prev. 39, 372–379. doi: 10.1016/j.aap.2006.08.013, PMID: 17054894

[ref33] Hayes A. F. Rockwood N. J. (2017). Regression-based statistical mediation and moderation analysis in clinical research: observations, recommendations, and implementation. Behav. Res. Ther. 98, 39–57. doi: 10.1016/j.brat.2016.11.001 27865431

[ref34] Hirth V. A. Davis B. Fridriksson J. Rorden C. Bonilha L. (2007). Cognitive performance and neural correlates of detecting driving hazards in healthy older adults. Dement. Geriatr. Cogn. Disord. 24, 335–342. doi: 10.1159/000108606, PMID: 17878731

[ref35] Just M. A. Keller T. A. Cynkar J. (2008). A decrease in brain activation associated with driving when listening to someone speak. Brain Res. 1205, 70–80. doi: 10.1016/j.brainres.2007.12.075, PMID: 18353285 PMC2713933

[ref36] Kan K. Schweizer T. A. Tam F. Graham S. J. (2013). Methodology for functional MRI of simulated driving. Med. Phys. 40:012301. doi: 10.1118/1.4769107 23298106

[ref37] Kim S. Ulfarsson G. F. (2004). Travel mode choice of the elderly: effects of personal, household, neighborhood, and trip characteristics. Transp. Res. Rec. 1894, 117–126. doi: 10.3141/1894-13

[ref38] Klauer S. G. Guo F. Simons-Morton B. G. Ouimet M. C. Lee S. E. Dingus T. A. (2014). Distracted driving and risk of road crashes among novice and experienced drivers. N. Engl. J. Med. 370, 54–59. doi: 10.1056/NEJMsa1204142, PMID: 24382065 PMC4183154

[ref39] Knight M. J. Mccann B. Tsivos D. Couthard E. Kauppinen R. A. (2016). Quantitative T 1 and T 2 MRI signal characteristics in the human brain: different patterns of MR contrasts in normal ageing. MAGMA 29, 833–842. doi: 10.1007/s10334-016-0573-0, PMID: 27333937 PMC5124042

[ref40] Leversen J. S. Hopkins B. Sigmundsson H. (2013). Ageing and driving: examining the effects of visual processing demands. Transport. Res. F: Traffic Psychol. Behav. 17, 1–4. doi: 10.1016/j.trf.2012.11.003

[ref41] Lew H. L. Poole J. H. Lee E. H. Jaffe D. L. Huang H.-C. Brodd E. (2005). Predictive validity of driving-simulator assessments following traumatic brain injury: a preliminary study. Brain Inj. 19, 177–188. doi: 10.1080/02699050400017171, PMID: 15832892

[ref42] Li K. Z. Bherer L. Mirelman A. Maidan I. Hausdorff J. M. (2018). Cognitive involvement in balance, gait and dual-tasking in aging: a focused review from a neuroscience of aging perspective. Front. Neurol. 9:913. doi: 10.3389/fneur.2018.00913, PMID: 30425679 PMC6219267

[ref43] Lin C.-T. Chen S.-A. Chiu T.-T. Lin H.-Z. Ko L.-W. (2011). Spatial and temporal EEG dynamics of dual-task driving performance. J. Neuro Eng. Rehabil. 8:11. doi: 10.1186/1743-0003-8-11, PMID: 21332977 PMC3050807

[ref44] Litwin H. Levinson M. (2018). The association of mobility limitation and social networks in relation to late-life activity. Ageing Soc. 38, 1771–1790. doi: 10.1017/S0144686X1700023X

[ref45] Liu T. Saito H. Oi M. (2012). Distinctive activation patterns under intrinsically versus extrinsically driven cognitive loads in prefrontal cortex: a near-infrared spectroscopy study using a driving video game. Neurosci. Lett. 506, 220–224. doi: 10.1016/j.neulet.2011.11.009 22101357

[ref46] Lyon C. Brown S. Vanlaar W. Robertson R. (2021). Prevalence and trends of distracted driving in Canada. J. Saf. Res. 76, 118–126. doi: 10.1016/j.jsr.2020.12.005, PMID: 33653542

[ref47] Macdonald M. E. Pike G. B. (2021). MRI of healthy brain aging: a review. NMR Biomed. 34:e4564. doi: 10.1002/nbm.4564 34096114

[ref48] Maltz M. Shinar D. (1999). Eye movements of younger and older drivers. Hum. Factors 41, 15–25. doi: 10.1518/001872099779577282, PMID: 10354803

[ref49] Mayhew D. R. Simpson H. M. Wood K. M. Lonero L. Clinton K. M. Johnson A. G. (2011). On-road and simulated driving: concurrent and discriminant validation. J. Saf. Res. 42, 267–275. doi: 10.1016/j.jsr.2011.06.004, PMID: 22017829

[ref50] Meda S. A. Calhoun V. D. Astur R. S. Turner B. M. Ruopp K. Pearlson G. D. (2009). Alcohol dose effects on brain circuits during simulated driving: an fMRI study. Hum. Brain Mapp. 30, 1257–1270. doi: 10.1002/hbm.20591, PMID: 18571794 PMC2751645

[ref51] Meuleners L. B. Harding A. Lee A. H. Legge M. (2006). Fragility and crash over-representation among older drivers in Western Australia. Accid. Anal. Prev. 38, 1006–1010. doi: 10.1016/j.aap.2006.04.005, PMID: 16713982

[ref52] Murphy S. L. Xu J. Kochanek K. D. (2013). Deaths: final data for 2010. Natl. Vital Stat. Rep. 61, 1–117. 24979972

[ref53] National Center for Statistics and Analysis (2021). Traffic safety facts: Young drivers (technical report no. DOT HS 813 130) National Highway Traffic Safety Administration.

[ref54] Nieborowska V. Lau S.-T. Campos J. Pichora-Fuller M. K. Novak A. Li K. Z. (2018). Effects of age on dual-task walking while listening. J. Mot. Behav. 51, 416–427. doi: 10.1080/00222895.2018 30239280

[ref55] Nyberg J. Björklund G. Aretun Å. Berg H.-Y. Strandberg T. (2021). How does driving license withdrawal affect subjective well-being? A Swedish comparative survey study of visual field loss. Eur. Transp. Res. Rev. 13:51. doi: 10.1186/s12544-021-00511-4

[ref56] Ohata R. Ogawa K. Imamizu H. (2022). Neuroimaging examination of driving mode switching corresponding to changes in the driving environment. Front. Hum. Neurosci. 16:788729. doi: 10.3389/fnhum.2022.788729, PMID: 35250514 PMC8895376

[ref57] Oxley J. Whelan M. (2008). It cannot be all about safety: the benefits of prolonged mobility. Traffic Inj. Prev. 9, 367–378. doi: 10.1080/15389580801895285, PMID: 18696394

[ref58] Páez A. Scott D. Potoglou D. Kanaroglou P. Newbold K. B. (2007). Elderly mobility: demographic and spatial analysis of trip making in the Hamilton CMA, Canada. Urban Stud. 44, 123–146. doi: 10.1080/00420980601023885

[ref59] R Core Team (2020). "R: A language and environment for statistical computing". (Vienna, Austria: R Foundation for Statistical Computing).

[ref60] Rabbitt P. Carmichael A. Jones S. Holland C. (1996). When and why older drivers give up driving. London, UK: AA Foundation for Road Safety Research.

[ref61] Ranchet M. Broussolle E. Poisson A. Paire-Ficout L. (2012). Relationships between cognitive functions and driving behavior in Parkinson’s disease. Eur. Neurol. 68, 98–107. doi: 10.1159/000338264, PMID: 22759624

[ref62] Robertsen R. Lorås H. W. Polman R. Simsekoglu O. Sigmundsson H. (2022). Aging and driving: a comparison of driving performance between older and younger drivers in an on-road driving test. SAGE Open 12:215824402210961. doi: 10.1177/21582440221096133

[ref63] Romoser M. R. Pollatsek A. Fisher D. L. Williams C. C. (2013). Comparing the glance patterns of older versus younger experienced drivers: scanning for hazards while approaching and entering the intersection. Transport. Res. F: Traffic Psychol. Behav. 16, 104–116. doi: 10.1016/j.trf.2012.08.004, PMID: 23148130 PMC3494462

[ref64] Schultheis M. T. Manning K. J. (2011). “Neuroscience and older drivers” in Handbook of traffic psychology (Elsevier), 127–136.

[ref65] Schweizer T. Kan K. Hung Y. Tam F. Naglie G. Graham S. (2013). Brain activity during driving with distraction: an immersive fMRI study. Front. Hum. Neurosci. 7:53. doi: 10.3389/fnhum.2013.00053, PMID: 23450757 PMC3584251

[ref66] Scott D. M. Newbold K. B. Spinney J. E. Mercado R. Paez A. Kanaroglou P. S. (2009). New insights into senior travel behavior: the Canadian experience. Growth Chang. 40, 140–168. doi: 10.1111/j.1468-2257.2008.00464.x

[ref67] Shechtman O. Classen S. Awadzi K. Mann W. (2009). Comparison of driving errors between on-the-road and simulated driving assessment: a validation study. Traffic Inj. Prev. 10, 379–385. doi: 10.1080/15389580902894989 19593717

[ref68] Sherif T. Rioux P. Rousseau M.-E. Kassis N. Beck N. Adalat R.. (2014). CBRAIN: a web-based, distributed computing platform for collaborative neuroimaging research. Front. Neuroinform. 8:54. doi: 10.3389/fninf.2014.00054 24904400 PMC4033081

[ref69] Sleightholm M. Billette J. Normandin C. Hofmann N. (2010). The use of transportation by seniors in Canada. Environ. Stats. 4, 12–15.

[ref70] Thach W. T. Goodkin H. Keating J. (1992). The cerebellum and the adaptive coordination of movement. Annu. Rev. Neurosci. 15, 403–442. doi: 10.1146/annurev.ne.15.030192.002155 1575449

[ref71] Uchiyama Y. Ebe K. Kozato A. Okada T. Sadato N. (2003). The neural substrates of driving at a safe distance: a functional MRI study. Neurosci. Lett. 352, 199–202. doi: 10.1016/j.neulet.2003.08.072, PMID: 14625019

[ref72] Unni A. Ihme K. Jipp M. Rieger J. W. (2017). Assessing the driver’s current level of working memory load with high density functional near-infrared spectroscopy: a realistic driving simulator study. Front. Hum. Neurosci. 11:167. doi: 10.3389/fnhum.2017.00167, PMID: 28424602 PMC5380755

[ref73] Vonk J. M. Rizvi B. Lao P. J. Budge M. Manly J. J. Mayeux R.. (2019). Letter and category fluency performance correlates with distinct patterns of cortical thickness in older adults. Cereb. Cortex 29, 2694–2700. doi: 10.1093/cercor/bhy138, PMID: 29893804 PMC6519688

[ref74] Walter H. Vetter S. C. Grothe J. Wunderlich A. P. Hahn S. Spitzer M. (2001). The neural correlates of driving. Neuroreport 12, 1763–1767. doi: 10.1097/00001756-200106130-00049 11409755

[ref75] Westlye L. T. Grydeland H. Walhovd K. B. Fjell A. M. (2011). Associations between regional cortical thickness and attentional networks as measured by the attention network test. Cereb. Cortex 21, 345–356. doi: 10.1093/cercor/bhq101, PMID: 20525771

[ref76] Williams A. F. (2003). Teenage drivers: patterns of risk. J. Saf. Res. 34, 5–15. doi: 10.1016/S0022-4375(02)00075-0 12535901

[ref77] Woods-Fry H. Vanlaar W. G. Robertson R. D. Torfs K. Kim W. Van Den Berghe W.. (2018). Comparison of self-declared Mobile use while driving in Canada, the United States, and Europe: results from the European survey of road users’ safety attitudes. Transp. Res. Rec. 2672, 74–83. doi: 10.1177/0361198118787631

[ref78] Wynne R. A. Beanland V. Salmon P. M. (2019). Systematic review of driving simulator validation studies. Saf. Sci. 117, 138–151. doi: 10.1016/j.ssci.2019.04.004

[ref79] Young K. Regan M. Hammer M. (2007). Driver distraction: a review of the literature. Distracted driving 2007, 379–405.

